# Degradation mechanisms of perovskite light-emitting diodes under electrical bias

**DOI:** 10.1515/nanoph-2022-0569

**Published:** 2022-11-10

**Authors:** Dong Guang Zheng, Dong Ha Kim

**Affiliations:** Department of Chemistry and Nano Science, Ewha Womans University, 52, Ewhayeodae-gil, Seodaemun-gu, Seoul 03760, Korea

**Keywords:** degradation mechanisms, device structure, light-emitting diodes, perovskites

## Abstract

Metal-halide perovskite light-emitting diodes (PeLEDs) are considered as new-generation highly efficient luminescent materials for application in displays and solid-state lighting. Since the first successful demonstration of PeLEDs in 2014, the research on the development of efficient PeLEDs has progressed significantly. Although the device efficiency has significantly improved over a short period of time, their overall performance has not yet reached the levels of mature technologies for practical applications. Various degradation processes are the major impediment to improving the performance and stability of PeLED devices. In this review, we discuss various analysis techniques that are necessary to gain insights into the effects of various degradation mechanisms on the performance and stability of PeLEDs. Based on the causes and effects of external and internal factors, the degradation processes and associated mechanisms are examined in terms of critical physical and chemical parameters. Further, according to the progress of the current research, the challenges faced in studying degradation mechanisms are also elucidated. Given the universality of the degradation behavior, an in-depth understanding of the device degradation may promote the development of optimization strategies and further improve the performance and stability of PeLEDs.

## 1 Introduction

**Figure 1:**
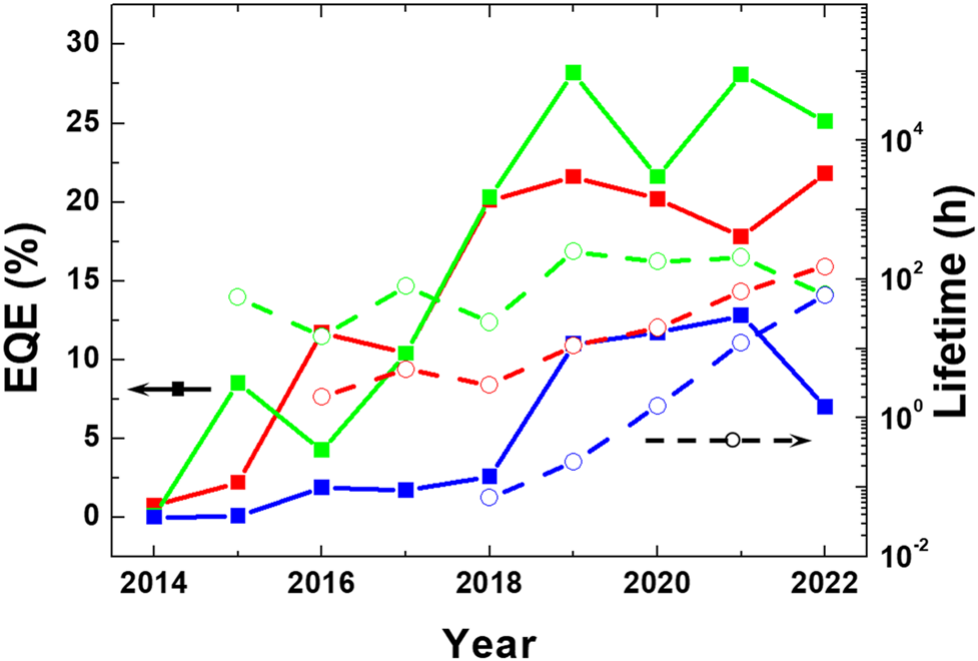
Overview of recent progress on the external quantum efficiency and lifetime of PeLED devices. The data are collected from previous studies and are tabulated in Table 1 [2], [3], [4], [5], [6], [7], [8], [9, 19, 20, 29, 34], [35], [36], [37], [38], [39], [40], [41, 54], [55], [56], [57], [58, 66], [67], [68], [69, 71, 73], [74], [75], [76, 79], [80], [81, 99, 130, 131, 157, 158, 167, 193].

Perovskites were first discovered by Gustav Pose in 1839 as the mineral called perovskite, which consists of calcium titanium oxide (CaTiO_3_), and was named after the Russian mineralogist L. A. Perovski. Materials with a similar type of crystal structure represented by the general chemical formula ABX_3_ can be described as perovskites. The use of metal halide perovskites (MHPs) in thin-film light-emitting diodes (LEDs) can be traced back to Mitzi and coworkers, who implemented this strategy in the 1990s [1], whereas room-temperature electroluminescence (EL) from perovskite LEDs (PeLEDs) was not demonstrated only recently in 2014 by Tan et al. [2]. To date, green and red PeLEDs have been rapidly developed with an external quantum efficiency (EQE) exceeding 20% [3], [4], [5], [6], [7], [8], [9]. Although the performance of blue PeLEDs is not yet comparable to their counterparts, the development of these devices has progressed at an excellent rate. The state-of-the-art development of performance and stability of PeLED devices over several years are summarized (for a more complete list, see Figure 1 and Table 1). Despite the promising commercialization in the form of lightings and displays, the applications of PeLEDs are still impeded by several issues related to their stability [10], [11], [12], [13], [14], [15], [16], [17], [18].

**Table 1:** Summary of the performance and stability of PeLEDs.

Device structure	Wavelength	EQE_max_	Stability	Year	Ref.
	(nm)	(%)			
ITO/TiO_2_/Al_2_O_3_/CH_3_NH_3_PbI_3–*x*_Cl_*x*_/F8/MoO_3_/Ag	630	0.76^L^		2014	[2]
ITO/TiO_2_/Al_2_O_3_/CH_3_NH_3_PbBr_3_/F8/MoO_3_/Ag	517	0.1^L^		2014	[2]
ITO/PEDOT:PSS/PVK/CsPbX_3_ (X = Br, Cl)/TPBi/LiF/Al	455	0.07^L^		2015	[33]
ITO/ZnO/CH_3_NH3PbI_3–*x*_Cl_*x*_/TFB/MoO_*x*_/Au	768	15.7		2015	[34]
SOCP/MAPbBr_3_/TPBi/LiF/Al	540	8.53^L^		2015	[147]
ITO/c-TiO_2_/EA/CH_3_NH_3_PbBr_3_/spiro-OMeTAD/Au	530	0.051^L^	55 h at 120 cd/cm^2^	2015	[67]
ITO/PEDOT:PSS/CsPbBr_3_/B3PYMPM/CsCO_3_/Al	524	0.15^L^	15 h at 66.7 mA/cm^-2^	2016	[41]
ITO/PEDOT:PSS/CsPbBr_3_/TPBi/LiF/Al	521	4.26^L^		2016	[35]
Au/ZnO/PEIE/NFPI_7_/TFB/MoO_3_/Au	763	11.7	2 h at 10 mA/cm^-2^	2016	[50]
ITO/PEDOT:PSS/PVK/CsPbX_3_ (X = Br, Cl)/TPBi/LiF/Al	490	1.9		2016	[54]
ITO/ZnO/Cs_10_(MA_0.17_FA_0.83_)_(100*-x*)_PbC_l1.5_Br_1.5_/α-NPD/MoO_3_/Au	475	1.7^L^		2017	[19]
Au/ZnO/PEIE/NCPbI_7_/TFB/MoO_3_/Au	689	3.7	5 h at 10 mA/cm^-2^	2017	[74]
ITO/ZnO/PVP/Cs_0.87_MA_0.13_PbBr_3_/CBP/MoO_3_/Al	520	10.4^L^		2017	[51]
ITO/PEDOT:PSS/CsPbBr_3_/TPBi/LiF/Al	525	4.76^L^	80 h at 1000 cd/cm^2^	2017	[158]
ITO/m-PEDOT:PSS/CsPbBr_3_/Bphen/LiF/Ag	524	0.9^L^	24 h at 100 cd/cm^2^	2018	[99]
ITO/PEDOT:PSS/CsPbBr_3_/MABr/B3PYMPM/LiF/Al	525	20.3^L^	104.56 h at 100 cd/cm^2^	2018	[3]
ITO/MZO/PEIE/PPBH/TFB-PFO/MoO_*x*_/Au	800	20.1^L^	46 h at 0.1 mA/cm^-2^	2018	[4]
ITO/AZO/AZO:Cs/Cs_*x*_(MA_0.17_FA_0.83_)_(100*-x*)_Pb(BrxI_1*-x*_)_3_/CuSGaSnO/WO_3_/Ag	475	2.58^L^	16 h at 1000 cd/cm^2^	2018	[167]
ITO/PEDOT:PSS/PEA_2_(MA/Cs)_1.5_Pb_2.5_Br_8.5_/TPBI/LiF/Al	490	1.5	0.17 h at 10 cd/cm^2^	2018	[40]
ITO/PEDOT:PSS/poly-TPD/CsPbBr_3_/TPBi/Liq/Al	645	14.1^L^	3 h at 100 cd/cm^2^	2018	[75]
ITO/ZnO:PEIE/FAPbI_3_/TFB/MoO_3_/Au	800	21.6	20 h at 25 mA/cm^2^	2019	[5]
ITO/ZnO/PEDOT:PSS/CsPbBr_3_/TPBi/LiF/Al	514	28.2		2019	[36]
ITO/PEDOT:PSS/PEACl:CsPbBr_3_/TPBi/LiF/Al	477	11^L^	2 h at 100 cd/cm^2^	2019	[102]
ITO/PEDOT PSS/PA_2_CsPb_2_I_7_/TPBi/LiF/Al	658	1.84	11 h at 3.72 W/sr m^-2^	2019	[81]
ITO/NiOx/TFB/PVK/CsPbBr_3_/TPBi/LiF/Al	483	9.5	0.07 hcat 100 cd/cm^2^	2019	[79]
ITO/PEDOT:PSS/FA_0.11_MA_0.10_Cs_0.79_PbBr_3_/TPBI/LiF/Al	518	17^L^	250 h at 100 cd/cm^2^	2019	[157]
ITO/PEDOT:PSS/PTAA/CsPbBr_3_/TPBi/PO-T2T/LiF/Al	520	21.6	180.1 h at 100 cd/cm^2^	2021	[6]
ITO/PEDOT:PSS:PFI/PEA_2_Cs_1.6_MA_0.4_Pb_3_Br_10_/TPBI/LiF/Al	479	5.2	1.5 h at 100 cd/cm2	2020	[66]
ITO/NiO_*x*_/PTAA/PVK/PEABr:CsPb(BrxCl_1*-x*_)_3_/TPBi/LiF/Al	488	11.7^L^	0.28 h at 100 cd/cm^2^	2020	[53]
Au/ZnO/PEIE/NMAFAPbI_7_/TFB/MoO_3_/Au	800	20.2		2020	[37]
ITO/poly-TPD/CsPbI_3_/TPBi/LiF/Al	689	14.8^L^	20 h at 100 cd/cm^2^	2020	[131]
ITO/m-PEDOT:PSS/(BTm)_2_SnI_4_/TPBi/LiF/Al	627	3.33	66 h at 95 cd/cm^2^	2021	[80]
ITO/PEDOT:PSS/poly-TPD/β-CsPbI_3_/TPBi/LiF/Al	689	17.8	317 h at 30 mA/cm^-2^	2021	[18]
ITO/poly-TPD/LiF/PEA_2_Cs_*n*−1_PbnBr_3*n*+1_/TPBi/CsF/Al	514	28.1	4.04 h at 100 cd/cm^2^	2021	[7]
ITO/PEDOT:PSS/p-F-PEABr&PEABr:CsPb(Br_*x*_Cl_1−*x*_)_3_/TPBi/LiF/Al	486	12.8	0.27 h at 150 cd/cm^2^	2021	[28]
ITO/PEDOT:PSS/PTAA/CsPbBr_3_/TPBi/PO-T2T/LiF/Al	520	21.6	180.1 h at 100 cd/cm^2^	2021	[6]
ITO/PVK:F4-TCNQ/PEA_0.5_CsPbBr_3.5_/TPBi/LiF/Al	518	16.8^L^	208 h at 100 cd/cm^2^	2021	[68]
ITO/PEDOT:PSS/CsPbBr_3_/TPBi/LiF/Al	470	4.7^L^	12 h at 102 cd/cm^2^	2021	[39]
ITO/NiMgLiO_*x*_/CsPbBr_3_/PMMA/B3PYMPM/LiF/Al	527	22.3	59.5 h at 130 cd/cm^2^	2022	[71]
ITO/PVK/PTAA/(PEA)_2_FA_4_Pb_5_Br_16_/TPBi/LiF/Al	530	25.1		2022	[8]
ITO/PEDOT:PSS/poly-TPD/KI/CsPbI_3_/TPBi/LiF/Al	687	21.8	1.15 h at 100 cd/cm^2^	2022	[9]
ITO/ZnO/Cs_0.2_FA_2.8_PbI_3_/TFB/MoO_*x*_/Au	787	18.2	151 h at 20 mA/cm^-2^	2022	[76]
ITO/PEDOT:PSS/CsPbBr_3_/TPBi/Yb/Ag	486	7^L^		2022	[52]
ITO/PEDOT:PSS/PVK/CsPbBr_3_/ZnO/Ag	469	5^L^	59.2 h at 100 cd/cm^2^	2022	[69]

^L^EQE calculated with Lambertian assumption.

To date, the reported major effects of different degradation processes on the performance and stability of PeLEDs are as follows [5, 19], [20], [21], [22], [23], [24]: (1) breakdown behavior of the device, (2) ion-induced device degradation, (3) degradation of optoelectronic characteristics, and (4) electrochemical reaction-induced decomposition. These effects can be attributed to different degradation mechanisms that arise from either extrinsic factors (process parameters, impurities, encapsulation, fabrication environment conditions) or intrinsic factors (the electrical or chemical reactions, charge carrier transport processes, self-heating behavior, or interfacial effects). The most common external causes for PeLED degradation are arguably environmental conditions and process parameters. Further, a large number of fabrication process-related degradation/failure mechanisms are well understood, and some of them can be avoided completely. The degradation processes that occur inside the PeLED structure due to different factors are more interesting. Under an electrical bias, the degradation of a perovskite layer and the generation of nonemissive areas reduce the device performance [25], [26], [27]; electrochemical processes at the interface damage the functional layer [18, 28, 29]; ionic activities in the perovskite may produce additional defects that degrade the performance of the PeLEDs [30, 31]; and invasion of impurities and migration of metal ions to the perovskite layer lead to irreversible degradation [32, 33]. In many cases, the effects of various degradation mechanisms may overlap and together determine the device degradation. Therefore, a detailed understanding of a single degradation phenomenon remains challenging. For this reason, various authors have proposed that degradation mechanisms should be investigated from various perspectives by using different analysis techniques, such as optoelectronic techniques, structure analyzing techniques, and electrochemical analysis techniques. In recent studies, the effect of degradation mechanisms on the performance and stability of PeLEDs has been reviewed along with improvement strategies based on the fabrication procedure and structure optimization [34], [35], [36], [37], [38], [39], [40], [41]. However, a lack of comprehensive understanding of the physical and chemical changes that occur inside the device structures as well as negligible information on the underlying mechanisms of degradation are the major drawbacks impeding further development of these devices. Thus, a systematic and comprehensive understanding of the degradation principle, process, and mechanism for device performance is still necessary.

In this review, we focus on the degradation processes that occur during the device operation and their associated mechanisms. The common analysis techniques are discussed, which are required to understand the device degradation caused by the changes in the physical and chemical properties of the PeLEDs. The presently available information about device degradation caused by external and internal factors is also provided. Understanding the degradation processes under diverse conditions will aid in exploring more feasible methods to suppress them and support the subsequent development of PeLEDs in terms of performance and stability.

## 2 Analytical techniques for perovskite LEDs

Analytical techniques are essential for evaluating the degradation/failure pathways within PeLEDs. A broad overview of the useful analytical techniques for evaluating the physical and chemical characteristics of PeLEDs is presented in this section. In many cases, the effects of various degradation mechanisms are superimposed and contribute together, and based on the individual strengths and limitations of each analytical technique, it is difficult to separately analyze the degradation mechanisms in different parts of the PeLED structure by standard accelerated lifetime tests. Thus far, various analytical techniques have been used to investigate the degradation processes and associated mechanisms of the different components of a PeLED structure.

### 2.1 Optoelectronic techniques and methods

The regular luminous–current–voltage (L–I–V) measurement is the basic characterization method for all organic and inorganic LED devices and is used to quantify the luminous efficiency, current efficiency, or EQE. The result of I–V measurements can be also fitted by the diode model to evaluate the series and parallel resistances as well as the reverse saturation current and ideality factor. This steady-state characterization carries substantial information about carrier injection, transport, and recombination processes that occur in the device structure and can be used to explain the degradation of optoelectronic characteristics due to device aging effectively [25, 31]. Electroabsorption spectroscopy provides the basic information about the built-in electrical field as well as mobile or fixed charge densities in the operating devices, and the built-in electrical field can be determined in specific layers [42, 43, 44]. Capacitance measurement in the frequency and time domain can distinguish and quantify between mobile ions and electronic defect states [45, 46]. The presence of trap states can be identified by thermally stimulated current measurements [11, 44, 47, 48]. Impedance spectroscopy is also a powerful technique for studying carrier trapping and transport in PeLEDs and provides information on the optoelectrical response in different timescales. With proper equivalent circuit models, including several passive elements, and based on reliable material parameters, information about trap states inside the layers, charge carrier transport, accumulation, and recombination at different interfaces can be extracted. In this case, a voltage is applied under different pulse durations to measure the current and capacitance transients. The difference timescales also allow an exclusive measurement of the electronic defect states via a very short pulse time for trap filling (capture). Analysis of capacitance rise and decay states can distinguish capacitance changes due to electronic defect states and mobile ions [46, 49]. In some cases, it is the most powerful method to quantify ion migration and identify the footprints of distinct mobile ion species. However, impedance spectroscopy requires an input alternating signal, whose amplitude is sufficiently large to obtain a smooth spectrum with negligible noise, but also small enough to not affect the electrical state of the device. The measurement frequency range is usually limited by the instrument and device. A longer measurement time in the low-frequency range indicates that the device instability may cause errors in the impedance spectrum and any subsequent fitting or analysis distortions.

Noncontact optical methods have been extensively applied to the characterization of PeLED devices. Infrared (IR) and Raman spectroscopy are two of the most widely used techniques for the determination of changes in the molecular structure. Due to the IR-nontransparent metal electrodes used in PeLED devices, the above methods have not been adapted for complete devices. The obvious temperature change during the device operation can be directly measured by an IR camera. With this method, the temperature distribution of the device surface can be measured to study the current crowding effect and thermal management. The Joule heating effect has been identified by many groups, but the actual temperature measurement result by this method needs to be further investigated [10, 50]. Optical microscopy is a popular method that uses visible light for directly evaluating the morphology, crystallization, multiphase structures, and fracture surfaces. However, this characterization method is restricted by the diffraction limit of visible light. Fluorescence, darkfield, and polarized optical microscopy techniques have been implemented to overcome this significant limitation of optical microscopy [51], [52], [53]. Further, other alternative types of microscopies may be used, such as scanning electron microscopy (SEM), transmission electron microscopy (TEM), scanning tunneling microscope (STM), and atomic force microscopy (AFM) [34, 54], [55], [56], [57], [58], [59], [60], [61], for obtaining images with higher spatial resolutions.

### 2.2 Imaging and mapping characterization techniques

Investigating the morphology can provide insights into different aspects of device degradation. For instance, AFM can be used to investigate the effectiveness of surface treatment and deposition characteristics through roughness change and degree of crystallization of the material (commonly single layers), which yields the morphology-related device lifetime [60, 62, 63]. However, some problems should also be considered, such as the single scan image size is small; the relatively slow scanning speed causes the thermal drift that affects the measurement of accurate distance; and inevitable image artifact due to unsuitable tip, a poor operation condition, or the device itself. STM can map ultra-high-resolution images at the atomic level and discern the atomic-scale properties of materials, including surface roughness, defects, and surface reaction mechanisms [61, 64]. STM is a specialized but fragile and expensive equipment, and the device operation requires a lot of skill and precision; otherwise, it can be difficult to use effectively. As a result, the effect of local changes on the optoelectronic properties can easily be probed. Secondary-ion mass spectrometry (SIMS) can be used to study the composition of solid surfaces and thin films by comparison against well-calibrated standards to obtain accurate quantitative results [65]. However, the reference material should be very similar to the studied sample and both of them must be compatible with ultra-high vacuum conditions. Analysis of some elements is impossible because the material sputtered from the surface may be consist of mono-atomic ions or molecular.

Both X-ray spectroscopy (XPS) and ultraviolet spectroscopy (UPS) can be used to determine the specific properties and composition of the device surface. They are standard methods for surface and interface evaluation. UPS has been widely used to determine the electronic structure of solid as well as surface-specific electronic states. It is often used to characterize the material to determine the work function [66, 67]. XPS is more commonly used than UPS and is often used to determine the elemental composition presented on the surface of the material. It also reveals contamination at the surface, chemical states, the empirical formula, and electronic states of the elements in a material [67], [68], [69]. Recently, XPS has been used to investigate the compositional change during perovskite degradation. This method has been employed to analyze the chemical states and molecular distribution of aged perovskite crystals [70]. Sample size may be a limiting issue for XPS measurements, and sample cutting may result in further contamination of the target region. The difference between the measured and real values is related to the relative inaccuracy during repeated investigations.

Various depth profiling techniques have been applied to PeLEDs investigation. Utilizing high-resolution X-ray photoelectron spectroscopy (HR-XPS) depth profiling technique individually detects single elements by the chemical shift of their binding energy. It also allows detecting chemical changes in the bonding conditions, quantitatively evaluating materials in separate layers, and thus identifying diffusion and migration mechanisms. Time-of-flight SIMS (TOF-SIMS) can be applied to identify the specific compound of molecules and absorbed contamination on the surface. It can detect all masses on material surfaces, surface morphology by lateral imaging, and depth profile information [3, 10, 71]. Auger electron spectroscopy (AES) is an emerging surface analysis technique that provides surface information on thin films, such as the elemental composition and chemical state, in the form of ultrahigh-spatial resolution elemental maps. From the measured intensity and kinetic energy of an Auger peak, the detected element can be identified and quantified. Scanning electron images with *in situ* AES mapping have been used to determine the elemental distribution of perovskite films with high spatial resolutions [72]. Depth profile information can also be obtained by scanning device cross sections with surface-specific methods, such as SEM or scanning ion microscopy (SIM).

### 2.3 Chemical analysis techniques

One of the main challenges of PeLED investigations is the effective analysis of chemical composition. X-ray diffraction (XRD) is a widely used technique to assess the crystallinity and structure of single-crystal or polycrystalline materials [73], [74], [75], [76]. Energy dispersive X-ray (EDX) spectroscopy is a well-known nondestructive X-ray technique that is used for elemental analysis or chemical characterization. EDX analysis is used on a large scale to obtain brief information about the elemental composition and to identify the presence of any impurity [77]. In particular, scanning transmission electron microscopy (STEM) combined with EDX spectroscopy has been employed to probe ion distribution in individual grains or in an entire PeLED. Variations in structure, composition, and morphology caused by ion motions can be well visualized by using STEM and EDX depth profiling. In operational PeLED devices, the depletion of Br-ions at the top surface of perovskite and their accumulation near the electron injection layer had been observed using cross-sectional STEM-EDX mapping [26]. The negative halide ions migrating toward the cathode side implies that the external electrical field is not the only driving force for ion movement, the internal electrical field formed by the gradient charge distribution due to charge injection imbalance and charge trapping also drives ion migration. The internal electrical field-driven Br-migration and I-diffusion into adjacent charge-transport layers had also been detected by EDX depth profiling [26].

Both grazing-incidence small-angle X-ray scattering (GISAXS) and grazing-incidence wide-angle X-ray scattering (GIWAXS) are extensively used to study the crystal structure and inner morphology. GISAXS is an excellent method to characterize nanoscale density correlations and/or the shape of nanoscopic objects at surfaces, at buried interfaces, or in thin films [78, 79]. GISAXS combines features from small-angle X-ray scattering and diffuse X-ray reflectivity, which provides information about both lateral and normal ordering at a surface or inside a thin film, and can be used to determine the average domain size of different phases and provides information on domain purity [80, 81]. GIWAXS is a related scattering technique probing crystal orientation, crystalline lattice spacing, crystalline correlation length (CCL), and relative crystallinity. It is closely related to grazing incidence diffraction for getting detailed crystallographic determination. Meanwhile, the morphology and microstructure correlations in nanoscopic systems can be monitored *in situ* and in real time, and thus the kinetics properties of the self-assembly can be studied by *in situ* GISAXS and GIWAXS experiments. The GISAXS and GIWAXS scattering geometries are straightforward and, in many cases, without the need for scanning, making them very attractive to combine with *in situ* chambers.

The PeLED degradation can be affected by various factors, such as process parameters, environmental factors, and external and internal factors. Further, two or more factors simultaneously impact the device leading to a more complicated situation. Only accurate characterization of PeLEDs analyzes properly the degradation processes and associated mechanisms. Analytical techniques can help researchers to obtain meaningful insights from a mass of measurement results. The analysis of measured quantities based on different analytical techniques is perhaps the most important component of degradation research. Weak analysis produces inaccurate and unfitted explanations for the degradation behaviors that not only affect the veracity of the research but also make the findings unusable. The trend of technological development is to comprehensively utilize different analytical techniques to quantitatively and qualitatively research and to elucidate the PeLED degradation.

## 3 Degradation modes related to defects

### 3.1 Defining the device lifetime

**Figure 2:**
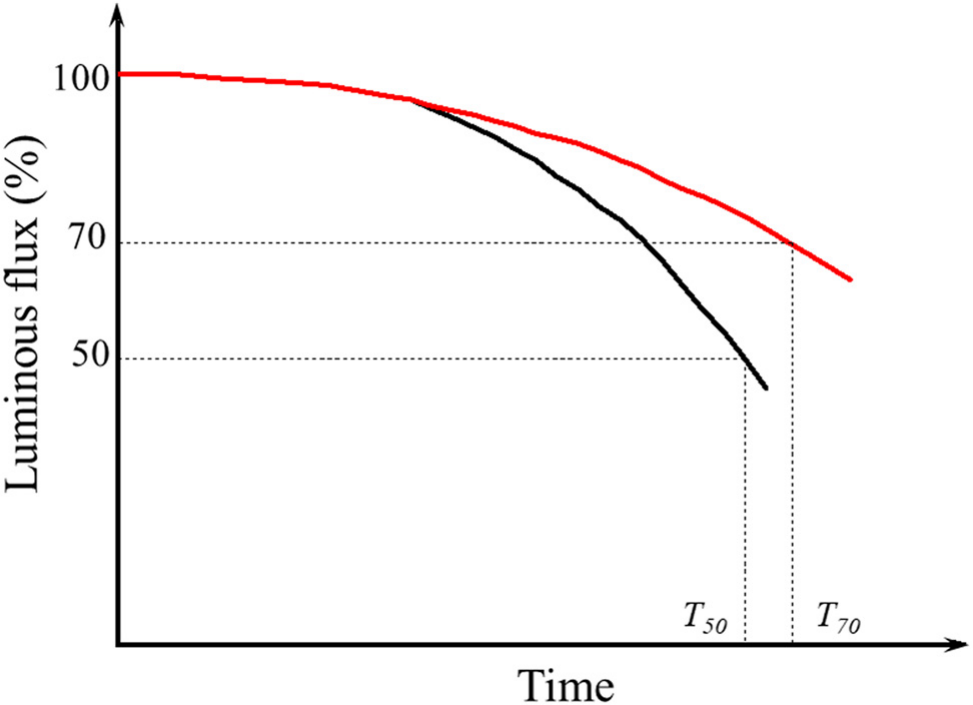
Lifetime estimation based on PeLED life testing.

The degradation usually implies that some processes lead to undesirable states in PeLED devices, such as chemical reactions and changes in morphological structure and physical properties. Various changes in device characteristics caused by device degradation usually manifest as device efficiency losses, with the degradation of L–I–V characteristics being the most obvious. Therefore, the evaluation of the lifetime of PeLEDs is mainly based on observing the time-dependent luminance parameter in a predefined physical environment. The device lifetime is defined as luminance maintenance, in which the brightness tends to diminish over time. The Alliance for Solid-State Illumination Systems and Technologies (ASSIST) defined the LED lifetime as the time in which the brightness drops to 50% (T50: for the display industry approach) or 70% (T70: for the lighting industry approach) of its initial brightness at room temperature as shown in Figure 2 [82]. Although there is a large discrepancy between the current lifetime and the actual minimum lifetime required, considering the short period of PeLED research, more progress can be made in improving the lifetime of PeLEDs. Some groups investigated device degradation from a chemical and physical perspective based on the L–T curve at different measurement conditions.

### 3.2 Relationship between degradation modes and associated mechanisms

**Figure 3:**
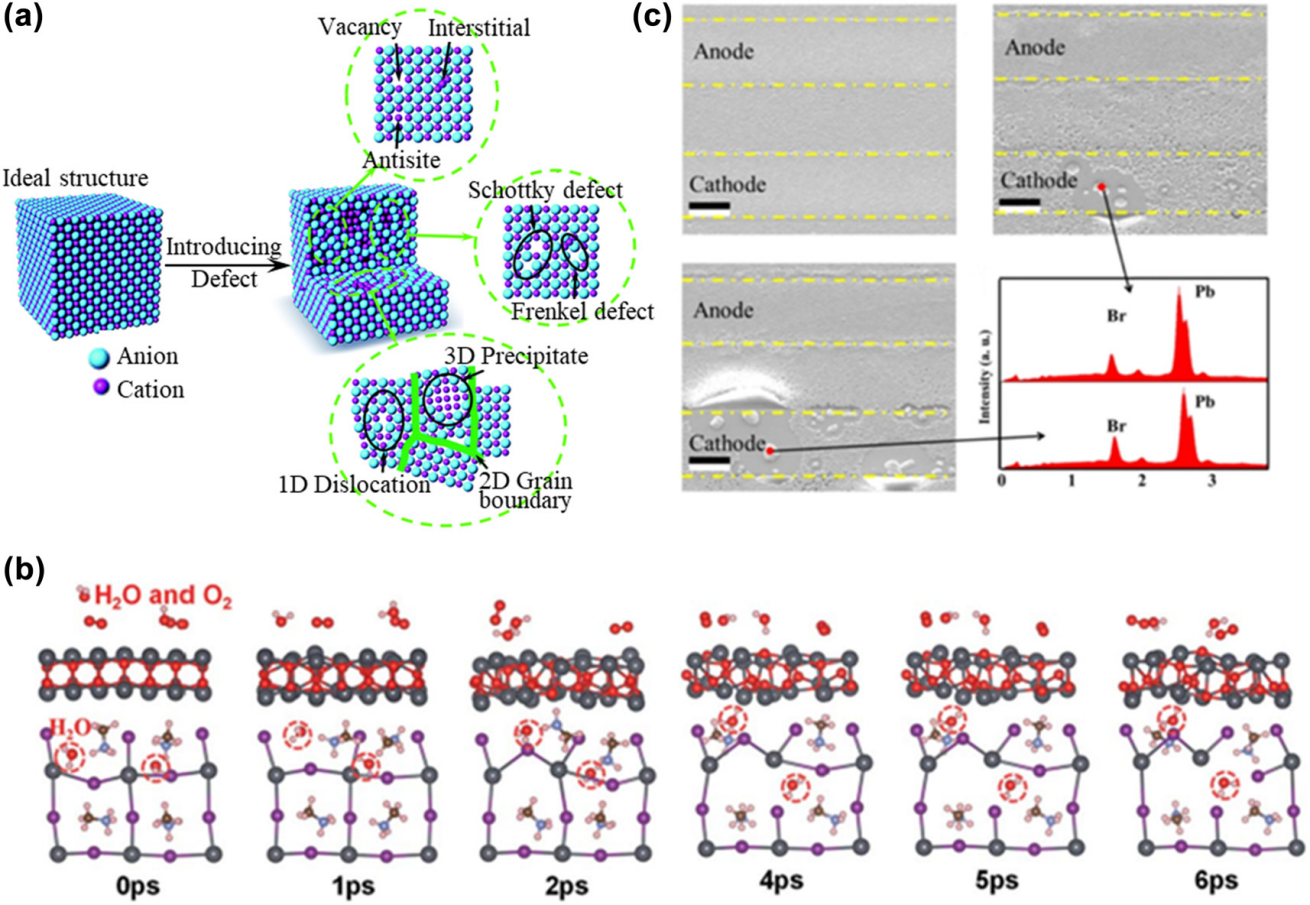
Defect types and defect effect on perovskite stability. (a) Illustration of an ideal crystal structure without defects and that after introducing defects, showing the microscopic configurations for different types of defects existing in the crystal lattice. Reprinted with permission from Ref. [16]. Copyright 2018 The Royal Society of Chemistry. (b) Corrosion due to moisture and superoxide formation in the crystal structure of MAPbI_3_. Reprinted with permission from Ref. [83]. Copyright 2019 The Royal Society of Chemistry. (c) The formation of element clusters caused by ion migration under an electrical bias. Reprinted with permission from Ref. [25]. Copyright 2018 Springer Nature.

Defects typically appear where the periodicity of the lattice is destroyed and exist in nearly all semiconductors at room temperature. There are many inevitable defects, as shown in Figure 3(a), due to the mixed electronic–ionic characteristics of PeLEDs [16]. In addition, many factors in fabrication processes can cause defects in PeLEDs, such as moisture and oxygen as shown in Figure 3(b) [83]. The device degradation under an electrical bias also induces new defects, such as those generated by ion migration as shown in Figure 3(c) [25]. These defects exist from microscopic to macroscopic levels in the device structure and play a critical role in physical properties. Some defects are considered harmless and even induce required electronic properties such as improvement of performance by using alkali-metal atom doping [84], in which the phase distribution is managed by doping the sodium ions to effective exciton energy transfer [57] or the structure and optical properties are optimized by Ni doping [85]. However, most defects are extremely detrimental to optoelectronic characteristics and reduce the performance and stability of PeLEDs [86, 87]. Therefore, an in-depth understanding of the defect behaviors of perovskites is essential for exploring the device degradation. However, the number of reported studies on defect control strategies exceeds those on degradation mechanisms caused by such defects. The formation and effect of defects are inherently obscure, and the defect status during device operation is also generally unclear.

We first introduce the defect-related degradation modes in PeLEDs. The defect-related degradation modes can be separated into rapid degradation (the immediate device breakdown) and gradual degradation (long-term degradation behavior of the device). Defect-induced electrical short circuit is responsible for rapid degradation. Gradual degradation may result due to the recombination-enhanced point defect responses as well as increased internal stress. Rapid degradation is the most undesirable failure mode that causes a complete destruction of the device. In most cases, the defect-induced electrical short circuits occur because of different reasons, including a localized heating effect and electromigration of the cathode metal [15, 88, 89]. The localized heating effect may lead to the formation of contact between the electrodes, destruct the internal layer stack system, and delaminate the cathode material. Such morphological changes may eventually lead to the failure of the entire PeLED [10, 11, 21]. Electromigration of the cathode metal is another mechanism that causes catastrophic failures during device operation, forming thin metal filaments that extend through the perovskite layer to the opposite electrode. The process description of gradual degradation is as follows: when nonradiative recombination occurs in a defect, which leads to a point-defect reaction and new point-defect generation; moreover, the new defects can also act as nonradiative recombination centers; the migration and condensation of point defects form defect clusters. The above actions are continuously repeated, resulting in a continuous defect generation. Therefore, gradual degradation is mainly associated with the formation of new nonradiative recombination sites.

The causes of gradual degradation can be categorized as extrinsic and intrinsic factors. For example, device process parameters, device assembly, and environmental conditions can be categorized as extrinsic factors. Ion migration, charge accumulation, electrochemical reaction, unbalanced carrier injection, etc. can be classified as intrinsic factors. Among them, improving the performance and stability of PeLEDs by mitigating intrinsic degradation processes is more challenging, because a complete understanding of the causes of degradation and their associated degradation mechanisms is required. In the following, we will discuss in detail the causes of degradation, degradation processes, and associated degradation mechanisms.

## 4 External processes induced degradation

Most problems related to external factors are common for PeLEDs of any color. Fabrication process parameters, including solution process (solvent selection and solution-processed), spin-coating process (spin speed, spin time and annealing), thermal evaporation, and device encapsulation techniques, have significant impacts on the stability and efficiency. Environmental factors, such as oxygen and moisture during fabrication processes, can also cause deterioration. The impact of temperature changes (environment temperature) on PeLEDs is well known. However, the thermally induced degradation mechanism is still poorly understood to deserve further investigation. Meanwhile, different experimental approaches or schemes for the same devices can lead to inconsistencies in degradation rates.

### 4.1 Influence of the process parameters

Various process parameters of PeLED fabrication have an often-underestimated effect on performance and stability. Chemically cleaned and oxygen plasma treatment of the indium tin oxide (ITO) substrate has a significant effect on improving pinhole density, hole injection, and stability of the ITO/hole transport layer (HTL) interface [90]. The rotation speed in the spin-coating procedure has an effect on the characteristics and morphology of perovskite layers. The moisture and oxygen content in an N_2_ glove box within the process chamber has a major effect on the PeLED device [91, 92]. Moreover, the annealing process is considered to be a critical step to improve the quality of the PeLED device, which may be related to an *in situ* moisture and oxygen desorption [93, 94]. It is generally believed that moisture in the preparation environment affects the nucleation and growth of perovskite films, which further affects the uniformity and defect density of the perovskite film. The existence of oxygen also directly accelerates the degradation of the perovskite and deteriorates the performance of the device. Moreover, oxygen reacts with the evaporated metal, resulting in oxidation during the evaporation process.

The polarity of the polar solvent commonly used has a significant effect on the luminescence properties and morphological characteristics of the perovskites. Due to the poor stability of perovskites in the polar solvent, the inherent ionic nature and low lattice energy make perovskite soluble in almost all polar solvents. The polar solvent first acts on the surface ligands and destroys the equilibrium, resulting in the obvious removal of the surface ligands of perovskite, forming new defects that destroy the light-emitting structure. The unavoidably used polar solvents during typical synthesis or purification processes result in loss of stability and structural integrity for the perovskites. In the synthesis of perovskite precursors, the nucleation centers for perovskite growth normally degrade in polar solvents to compromise device stability [95]. For example, N,N-dimethylformamide (DMF) greatly affects the stability of perovskite quantum dots (PQDs), leading to the formation of more defect states [96, 97]; the ligand mixture (oleic acid (OA) and oleylammonium-oleate) leads to the transformation of α-CsPbI_3_ perovskite nanocrystals (PNCs) into the δ-phase [98]; and the excessive polar solvents cause the desorption of ligands of CsPbX3 nanocrystals progressively, leading to the degradation of CsPbX3 nanocrystals [100, 101].

Because of the lack of a precise control over the nucleation and growth processes, solution-processed perovskites always contain various unexpected defects. The formation of defects is determined by the thermodynamics and kinetics of the nucleation, is related to the energy of the defect formation, and is significantly affected by the atomic chemical potential [102]. The structure of the internal defect is significantly affected by the formation conditions and the external environment. Although PNCs and perovskite films have similar defect types, such as microscopic defects and structural defects, this does not imply that both of them have the exact same origins of defects. Because of the unstable ligand layer on the surface of PNCs, controlling their dimension and surface integrity during post-treatments, such as purification and dilution, is a difficult task, and this results in the formation of surface defects [30, 99, 101]. In contrast, spinning the perovskite precursor solution on a substrate to fabricate a perovskite film causes the perovskite film to undergo more complicated crystallization and growth processes during the spin-coating process [103]. Therefore, various defects, such as pinholes, point defects, and grain boundaries, are inevitable in the preparation of perovskite films. Although the formation mechanism of defects is possibly different, these defects have an important influence on the performance and stability, whether in PNCs or perovskite films.

The annealing process is considered critical for improving the quality of the perovskite film in terms of morphology and grain size [94, 104]. Improving the thermal stability is good for suppressing the formation of halide-related defect states in perovskites [81]. However, an excessive annealing temperature results in the loss of surface ligands, and thus, formation of surface defect states, which cause photoluminescence (PL) quenching [91, 92, 105]. The grain size of the CsPbBr_3_ nanocrystals remains constant in a certain annealing temperature range and increases significantly when the annealing temperature continuously rises, accompanied by an obvious PL degradation [106]. The hybrid perovskite MAPbI_3_ can degenerate to PbI_2_ during thermal annealing at a high temperature [107].

The sensitivity of the perovskite material to environmental factors emphasizes the requirement of encapsulation to obtain a stable lifetime. Rapid degradation of PeLED devices without encapsulation has been reported by many research groups [108, 109]. Encapsulation of PeLED devices usually involves covering the device with a thin glass coverslip, which is then sealed with UV curable epoxy resin to retard the penetration of moisture and oxygen. However, the encapsulation technique cannot reduce the effects of light and heat under operating conditions.

### 4.2 Moisture and oxygen induced degradation

**Figure 4:**
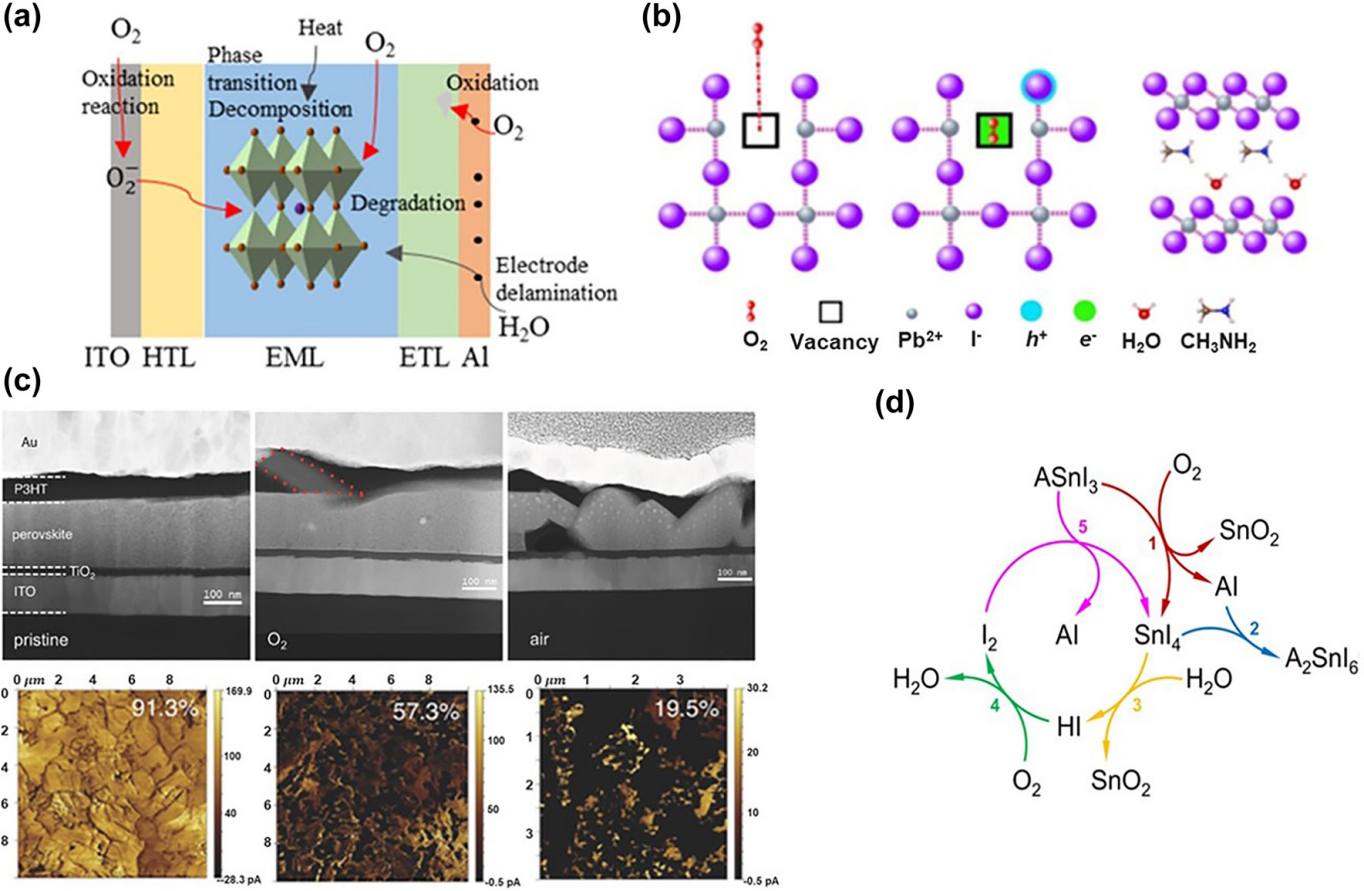
Moisture and oxygen induced device degradation. (a) Illustration of extrinsic stability under environmental conditions. (b) Schematic representation of the degradation processes caused by O_2_ inside a perovskite layer and substances produced by the reaction. Reprinted with permission from Ref. [115]. Copyright 2018 Springer Nature. (c) High-angle annular dark-field images (cross-sectional view) and AFM images of perovskite layers aged for 24 h in different atmospheres. Reprinted with permission from Ref. [92]. Copyright 2015 WILEY‐VCH Verlag GmbH & Co. KGaA, Weinheim. (d) SnI_4_ in tin iodide perovskites leads to device degradation by reacting with O_2_ and H_2_O. Reprinted with permission from Ref. [17]. Copyright 2019 Springer Nature.

The role of environmental factors cannot be ignored in the degradation of PeLEDs. Notably, perovskites are intrinsically highly sensitive to environmental factors due to their ionic nature. Figure 4(a) shows that the degradation processes initiated and participated by environmental factors induce the generation of defects in PeLEDs, which ultimately causes device degradation. Moisture and oxygen can penetrate into the device through defects at the edges of a metal cathode, resulting in chemical reactions with the metal cathode and degrading further the defect region. Some organic cations are sensitive to moisture and oxygen, causing the hydrolysis and oxidation of organic materials and cause irreversible degradation and decomposition.

The effects of moisture and oxygen on PeLEDs are a controversial topic. Many research groups have reported that the existence of moisture and oxygen has both positive and negative effects on perovskite devices. Moisture could be involved in the synthesis process to passivate defects [110, 111]. Moisture acted as an additive to promote the nucleation and crystallization of perovskite [112, 113]. During the annealing process, moisture assisted to suppress defect generation [114]. However, moisture can easily penetrate into the device through defects and participates in electrochemical reactions under operation at the organic/cathode interface, resulting in delamination of the metal layer. In addition, moisture-induced reactions also occur inside the perovskite layer, as shown in Figure 4(b) [115]. To hybrid perovskite, some organic cations, such as MA^+^ and FA^+^, were extremely sensitive to moisture, and moisture passivation often had a negative effect to degrade PeLEDs performance [116, 117]. With enough moisture penetration, MAPbI_3_ produced excess MAI and PbI_2_ leading to perovskite crystal decomposition [118, 119]. The duration of moisture treatment also affects the quality of perovskite films. A prolonged moisture treatment caused the grains to agglomerate and shrink, reducing film coverage [120].

Several researchers have reported that oxygen atoms reduced the hydrogen bonding ability of amino groups at molecule ends, increased the interaction probability with defects, reduced the density of traps for charge carriers, and enhanced light-emitting efficiency [121, 122]. Furthermore, oxygen can act as passivation molecules to strongly bond with the organic–inorganic hybrid perovskite at the defect sites, effectively inhibiting nonradiative recombination [11, 123]. However, many groups have also emphasized that oxygen causes perovskite instability. Oxygen is also an initiator for the degradation process, leading to the PeLED degradation and decomposition. Rapid diffusion of oxygen into the perovskite film is accompanied by a superoxide formation, which causes oxygen-induced perovskite degradation as shown in Figure 4(c) [92]. The existence of oxygen can cause the trapped charge in perovskites to weaken the Pb–I bond while strengthen the I–O and Pb–O bonds [124]. The rapid diffusion of oxygen into a perovskite by halide vacancies mediated occurred coordinates with electron-donating molecules through Lewis acid–base interactions [125]. The oxide transport material, such as TiO_2_, also indirectly promotes halide ion segregation in the perovskite film [126]. For hybrid organic–inorganic halide perovskites, oxygen is not only used as a passivation agent to enhance the PL emission but also directly accelerates the device degradation and deteriorates the device performance. As shown in Figure 4(d), based on the moisture and oxygen under ambient conditions, an Sn-based perovskite undergoes hydrolysis and oxidation reaction with moisture and oxygen to form I_2_, which participates in the reaction to generate more SnI_4_, leading to a cyclic degradation [17].

### 4.3 Impurity-induced degradation

The impurity in PeLED devices is considered to have a major effect on the performance and stability. Extrinsic impurities may promote metal electrode dissociation, and thus, metal atoms diffuse into the perovskite layer as new impurities and potentially cause device degradation. Intrinsic impurities may introduce deep levels that act as nonradiative recombination centers, compensate the built-in electrical field, change the band offset at the interfaces, and create shunting pathways. In addition, for quasi-2D PeLEDs, the existence of phase impurities may play a crucial role in seeding the device degradation.

Doping ABX_3_ perovskites is an effective strategy to develop PeLEDs; in this method, appropriate impurity elements are intentionally incorporated into the crystal lattices to achieve specific properties and functions. Several strategies involving A-site dopants have been shown to improve the stability due to suppression of ion transport [73, 127]. A partial B-site doping is also advantageous for the device performance and stability; for example, in a previous study, manganese (Mn^2+^) ions were incorporated into the crystal lattice to replace Pb^2+^ ions, resulting in lattice contraction, reduced nonradiative recombination, and an enhanced luminance efficiency [128]. Further, X-site doping has remained the main strategy to develop various-color PeLEDs [129, 130]. However, inappropriate doping also leads to defect density increases, optoelectronic properties reduced, and device stability decreases.

### 4.4 Thermal induced instability

Temperature-induced device degradation is one of the dominant external factors. Increase in ambient temperature not only affects the initial I–V–L characteristics of a device but also accelerates device degradation and even induces device failure at the critical temperature. With the increasing temperature, the thermally activated channels contribute to nonradiative recombination, and the light emission intensity exhibits a decreasing trend. In previously reported study on 2D/3D perovskite film properties, destruction of the 2D perovskite overlayer as well as the formation of an additional mixed phase at the interface accelerated the dark aging of the emission features under thermal stress [15]. PeLED devices need to satisfy the potential application conditions, which include a wide range of different environmental conditions and temperatures. Therefore, these devices should exhibit suitable temperature stability at least in the higher temperature range. Although several groups have studied the device performance and stability at increased temperatures [81, 105, 131], none of the work shown this far has been satisfactory.

### 4.5 Effect of measurement methods

**Figure 5:**
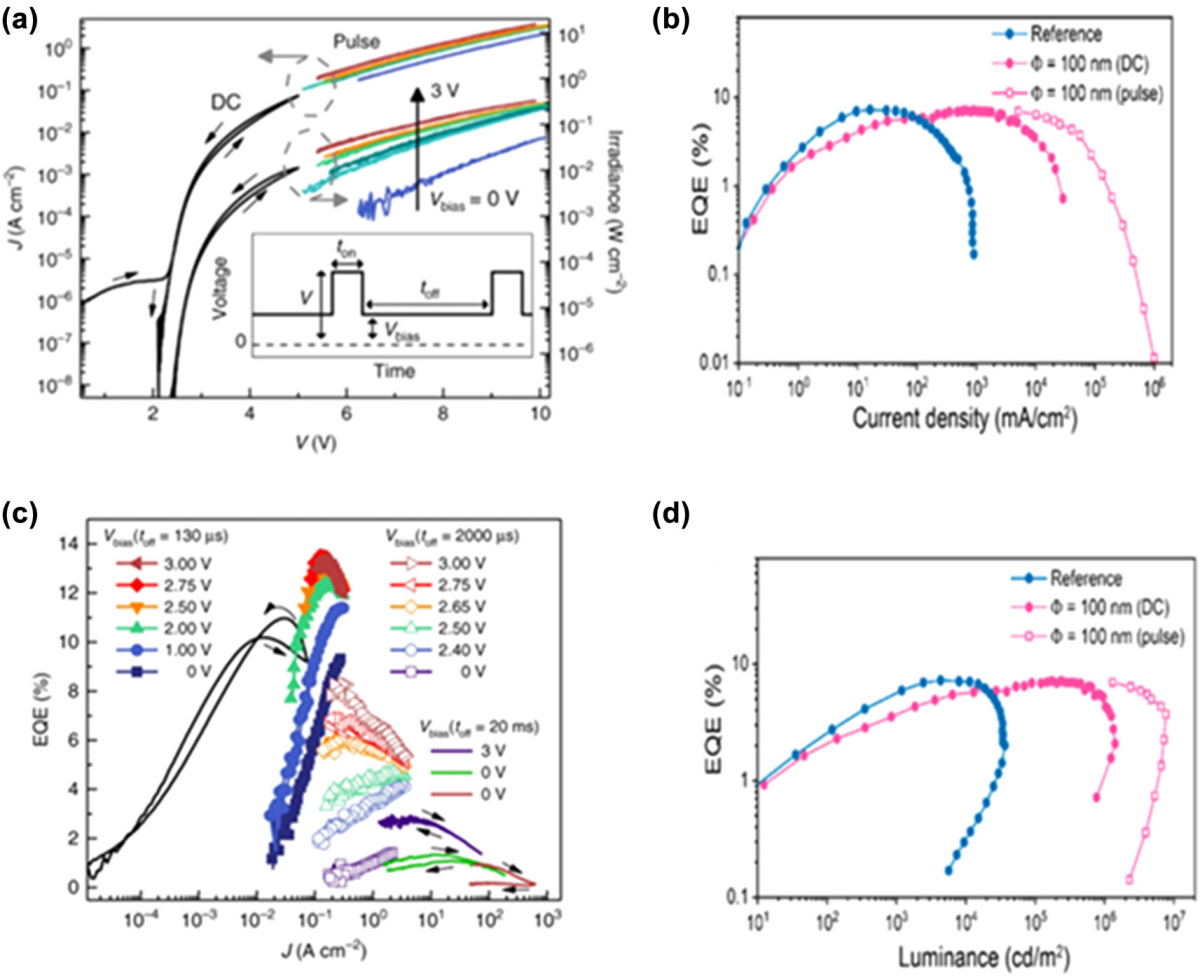
Optoelectronic characterization under DC and pulsed driving voltages. (a) And, (c) for the same device with varying pulse conditions. Reprinted with permission from Ref. [89]. Copyright 2019 Springer Nature. (b) And, (d) for nanopatterned PeLEDs driven by DC and pulsed currents. Reprinted with permission from Ref. [50]. Copyright 2019 American Chemical Society.

In order to study the degradation processes of PeLEDs, the effect of different driving schemes on device aging must be considered. Despite some advances in the reversibility of aging processes, systematic investigations of these effects on the PeLED operation are still lacking. For PeLEDs, the positive influence of the AC/pulse driving scheme is known, which causes back-diffusion of the ions, enhances the device stability, and exhibits a longer lifetime. The accumulation of charge carriers at the internal interfaces is one of the dominant mechanisms causing the degradation of PeLEDs. Using AC/pulse driving may mitigate this drawback towing to the release of the accumulated charges under the reverse bias condition [69]. Detailed studies on PeLEDs driven at the same luminance showed that these devices exhibit a longer lifetime behavior when driven at AC/pulse than at DC potentials [132, 133]. The electrical field-dependent mobile ion migration model can be used to describe and fit the voltage dependence of the forward and backward scan directions to analyze the ionic transport characteristics [89]. The monitored luminance exhibits only a minor change and a longer lifetime at the driven AC/pulse voltage, but the luminance shows a continuous decay. As shown in Figure 5(a) and (c) [89], the AC/pulse driving can contribute to long-term stability by mitigating the degradation processes inside the PeLEDs. AC/pulse driving only delays the luminance decay and does not prevent it from occurring as shown in Figure 5(b) and (d) [50].

## 5 Internal processes induced degradation

In contrast to extrinsic degradation factors, intrinsic degradation mechanisms are more difficult to study. In many cases, physical and chemical effects overlap and contribute together to determine the degradation processes of PeLEDs. It is difficult to obtain detailed information on the single degradation process. Therefore, it is important to design specific device structures, develop experiment procedures, and apply any available analysis techniques for elucidating the effects of specific internal factors, which pinpoint the crucial internal degradation processes that cause luminance inefficiency and device instability. The gradual degradation mechanism acts as secondary lifetime limiting process compared to the electrical overstress events that can induce sudden and catastrophic failure of devices.

The intrinsic degradation induced by an electrical bias during the device operation is related to the intrinsic properties of the PeLEDs. To understand the intrinsic degradation processes in more detail, here, we present the physical and chemical properties and summarize the different degradation mechanisms proposed to date, provide guidelines to aid in the analysis of the results, and highlight the progress made in improving the operational stability of these devices.

### 5.1 Degradation processes related to diffusion and drift mechanisms

The electrically neutral entities in perovskite layers can serve as nonradiative recombination centers or exciton quenchers. Ions also drift under an applied voltage or a built-in electrical bias. Moreover, the Joule heating effect also enhances metal diffusion into the perovskite layer.

Thin LiF layer is commonly used to enhance the performance of PeLEDs, where the LiF layer is deposited between the electron transport layer (ETL) and the cathode to enhance electron concentration in the ETL, to achieve balance between electron and hole injection for improving current efficiency, luminance efficiency, and EQE, effectively reducing the Schottky barrier height and width to decrease the effective turn-on voltage. However, during the process of the metal Al onto the LiF film by thermal evaporation, Al reacts with LiF, resulting in the dissociation of LiF. Since the Li migration process must occur on Li cations rather than Li atoms, the Li cations migration may be a diffusion process driven by the concentration gradient. Atomic Li would react with electron transport material to be reduced, and there is a propensity to bind to a metal Al ion to form Li:Al alloy. As a result of the strong ionic character of perovskites, ion migration is a known degradation pathway. Dissociated LiF under an electrical bias can migrate to the ETL or even the perovskite layer, thus generating defective leakage paths. Researchers have demonstrated the dissociation of the LiF layer after operation through various measurements, such as XPS [32], high-resolution electron energy loss spectroscopy [134], SIMS [135], and infrared reflection–absorption spectroscopy (IRRAS) [136]. Moreover, the insulator property of the LiF layer plays a dominant role in hindering the electron injection with the increase in the thickness of the LiF layer. The unbalanced charge carrier injection leads to charge carrier accumulation at the interface, resulting in the recombination zone shift toward the interface between the perovskite layer and ETL, and eventually affects the performance and stability of PeLEDs.

When ion migration begins from the perovskite layer, the formation of defective species and complexes induce interstitial states and accelerate the degradation of PeLEDs. Ion migration processes can be mainly divided into (1) ion migration within a grain in the perovskite layer induces the formation of defective pathways and enhances leakage current [137, 138]; (2) ion migration along or across grain boundaries leads to the accumulation of ions at the interface and increases the interface defect density [139]; and (3) ion migration across device interfaces results in changes in the charge carrier transport and carrier injection properties [140].

**Figure 6:**
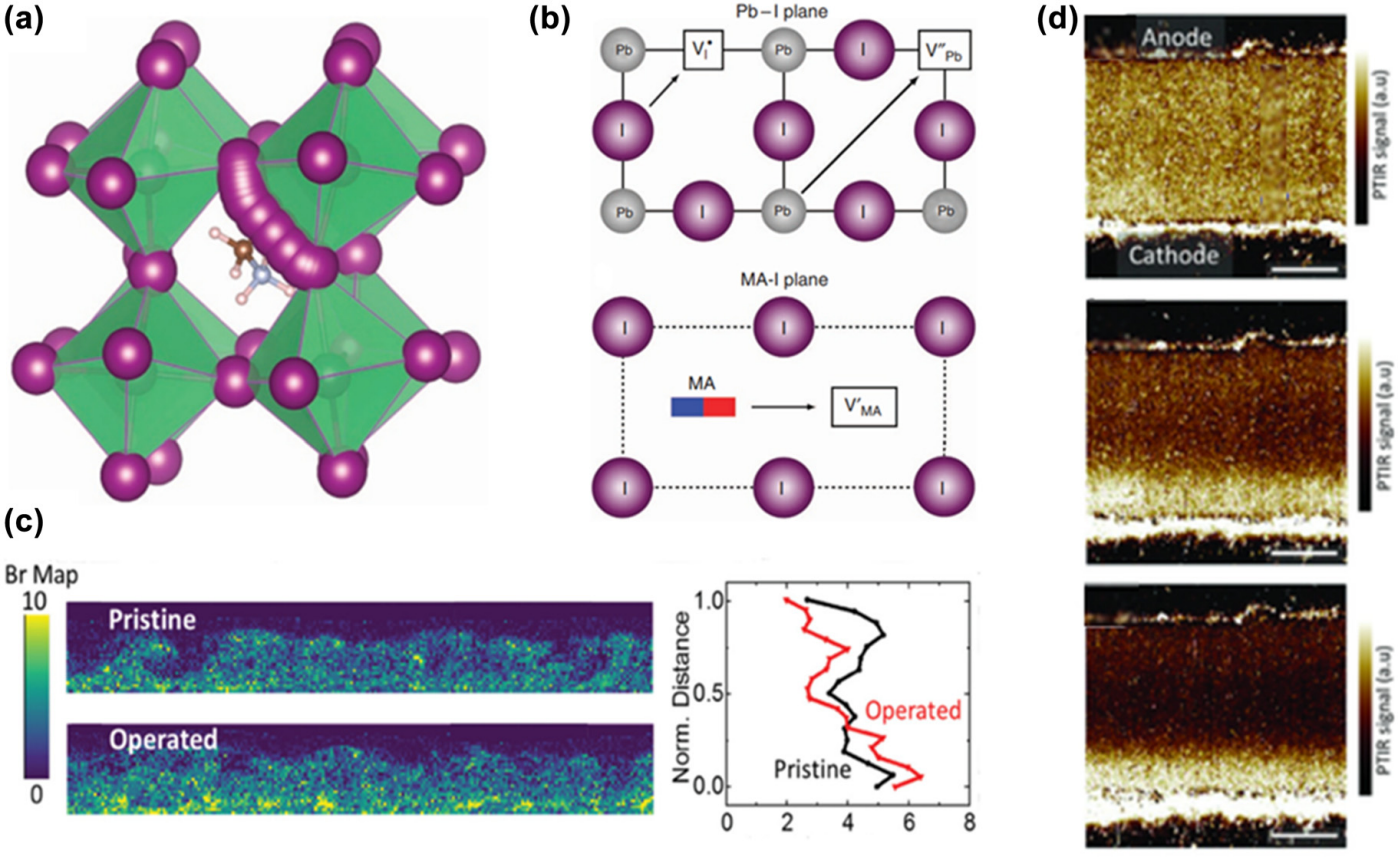
Ion migration path and ion redistribution in perovskite layer. (a) Migration path of I-ions in the MAPbI_3_ crystal was calculated by the density functional theory (DFT) method. (b) Illustration of the migration paths and activation energies. Panels a and b reproduced with permission from Ref. [142]. Copyright 2015 Springer Nature. (c) STEM-EDX maps of Br distribution in a pristine device and after operation at 2 V; the right panel shows the averaged intensities for Br through the normalized thickness of the film. Reproduced with permission [26]. Copyright 2020, Wiley‐VCH GmbH. (d) Schematic illustration of the PTIR images of the MAPbI_3_ before and after (100 s and 200 s) electrical poling, respectively. Reproduced with permission from Ref. [145]. Copyright 2015 WILEY‐VCH Verlag GmbH & Co. KGaA, Weinheim.

Perovskites have the general formula of ABX_3_, where the A-site ions are occupied by a monovalent cation, possibly organic or inorganic elements; the B-site ions are usually occupied by a divalent metal cation, commonly lead or tin; and the X-site ions are occupied by halide anions. Size mismatch between the ions may cause defects and distortions, which may affect the final decomposition of the crystal structure. Many defects in the perovskite layer can mediate ion migration under an electrical bias. Under a certain electrical bias, doping a certain number of cations (Cs^+^ and Rb^+^) at the A-site, the significant phenomena of ion migration could be observed [141]. The halide anions moved to the negative electrode via the halide vacancies, while the mobile interstitial Cs^+^ and Rb^+^ cations migrated to the positive electrode. First-principle calculations of MAPbI_3_ and MAPbBr_3_ perovskites indicate that the low activation energy of the halide vacancies and interstitial defects assist the ion migration along with the perovskite crystal as shown in Figure 6(a) [142], [143], [144]. The activation energy of iodine vacancies and interstitials are as low as 0.1 eV [144], and the subsequent activation energies of MA and Pb vacancies increase to ∼0.5 and 0.8 eV, respectively, as shown in Figure 6(b) [142]. Moreover, the grain boundaries on the surface provide stable sites for defects under the excitation of an electrical bias, and defect migration leads to halide segregation. The mixed halide phase separates into two individual halide phases as shown in Figure 6(c), and the emission peak of the mixed-halide perovskite shows red- or blue-shift [26].

Defects migrate along perovskite crystal and become trapped at the grain boundaries, due to the smallest potential energy of defects at the grain boundaries. Figure 6(d) presented the electromigration evidence of MA^+^ ions toward the cathode [145]. This also implied that the passivation of interface is useful in inhibiting the diffusion of defects, thus improving the performance and stability of PeLEDs [146, 147]. The original random distribution of ions moves directionally under an electrical bias, and the accumulated cations and negative charge centers form an extra built-in electrical field, which is favorable for the injection of both holes and electrons. However, ion migration induced gradual loss of defect passivation around the recombination zone in perovskite domain and thus resulted in EQE decay [148]. The accumulation and migration of defects and ions at the interface cause interface degradation. With operation time increased, a large number of mobile cations penetrated into the transport layer [21, 148, 149] and even could migrate to the electrode interface [25].

**Figure 7:**
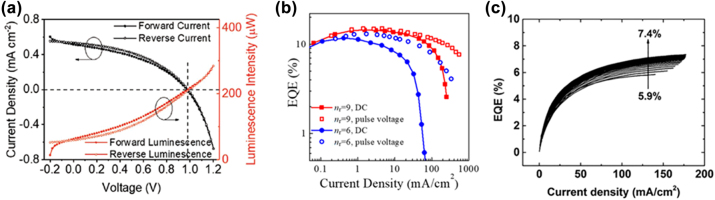
Impact of ion migration on PeLED operation. (a) Current density–voltage–luminescence intensity curves in the forward and reverse voltage scans. Reproduced with permission from Ref. [150]. Copyright 2020, Wiley‐VCH GmbH. (b) EQE versus current density of PeLEDs under DC and pulse operations. Reproduced with permission from Ref. [148]. Copyright 2020, American Chemical Society. (c) EQE versus current density of an as-produced perovskite LED for subsequent voltage scans. Reproduced with permission from Ref. [24]. Copyright 2017, WILEY‐VCH Verlag GmbH & Co. KGaA, Weinheim.

In Figure 7(a), the observed significant hysteresis in the I–V characteristics between the reverse and forward voltage scans indicates that the presence and redistribution of mobile ions by the electrical bias could weaken the built-in electrical field, thereby facilitating recombination in the perovskite layer [150]. An appropriate driven condition can reverse ion migration [89], resulting in the redistribution of mobile ions and the associated field confining the charge carriers near the interface between the perovskite layer and the transport layer. Therefore, radiative bimolecular recombination occurs more uniformly and efficiently throughout the perovskite layer as shown in Figure 7(b) [148]. Due to the trap-filling effect of the mobile ions in the perovskite layer, the subsequent voltage scans caused the EQE increase gradually in Figure 7(c) [24]. Although the redistribution of mobile ions can enhance the luminance efficiency, under continuous operation or high electrical bias, excess carrier injection can neutralize vacancies, weaken the attraction between vacancies and ions, and thus aggravate ion migration. Ions accumulation at the interface increases the lattice strain, which induces lattice distortion and atomic bond breakage [151, 152]. Ions penetration and trapping in the charge transport layer (CTL) or the interactions between mobile ions and CTL molecules destroy the properties of CTL, which induce extra band bending to inhibit the carrier injection and increase the voltage. The more significantly unbalanced carrier injection leads to the gradual approach of the recombination zone to the CTL side, enhancing the opportunity for excitons to be quenched by interface traps [14, 15, 25]. Meanwhile, the metal electrode can also be diffused into ETL to react with halide ions migrated from the perovskite layer, forming insulating metal halide complexes [152], [153], [154, 156].

### 5.2 Degradation processes related to nonradiative recombination centers

Nonradiative recombination is commonly related to the presence of defects. The excited carriers are captured by the defect sites and then relax gradually toward the ground state via phonon emission, causing severe trap-mediated nonradiative losses, and thus, a low emission efficiency. As well known, various defect generation is inevitable during crystallization due to the ionic character of the perovskite. Especially, to solution-processed PeLEDs, perovskite films tend to form defects with under-coordinated halide or metal ions at the surfaces and the grain boundaries, which act as the nonradiative recombination center at the interface of perovskite layer and transport layer [157, 158]. In addition, the formation of halide vacancies at the surface and grain boundaries is also ineluctable during the thermal annealing process. Moreover, the device operation may result in different degradation processes that may accumulate different reaction species, which are products of chemical reactions or migrating ions. These species result in a loss of excitons through nonradiative recombination and luminescence quenching [159]. Recent research has proved that trap states related to nonradiative recombination can be found in nanoscale clusters, and these defect states usually form at the junctions between the grains [160]. Most of the inherent point defects in perovskites form shallow carrier traps, which in turn generate recombination centers in the bandgap. These recombination centers trap the free charges and change the carrier recombination paths [159]. The charge trapping at the shallow-level defects may cause a certain amount of energy loss, resulting in a red-shifted radiative recombination. Defect trapping also has an impact on the corresponding quantum yield and carrier lifetimes.

According to several systematic studies on PeLED performance and stability, defects as nonradiative recombination centers can explain the inefficiency and instability of the PeLEDs. The degradation processes may initiate at the interface between the perovskite layer and the transport layer because of ions and defects that move toward the interface [27, 148]. The charge trapped at the interface between the perovskite and the charge transfer material generates an extra electrical field, which reduces the defect passivation and triggers decomposition of the perovskite layer [161]. The accumulation of mobile ions and increase in the defect states at the interface degrade the PeLEDs. Thus, minimizing defects is crucial for enhancing the radiative yield.

### 5.3 Role of charge carrier transport

Charge carrier trapping is unavoidable during carrier transport processes and has a significant impact on the device efficiency. The formation of different crystalline structures and amorphous phases affect the charge carrier transport and density of trap states. The traps (density and distribution, both energetic and spatial) could be formed by structural defects, chemical impurities, dipoles, excimers, dopants, or impurities [102, 162, 163]. The degradation processes of the PeLEDs also promote the formation of new traps, which in turn further aggravate the degradation of PeLEDs. Charge carrier trapping usually limits the charge carrier transport properties [164], affects the recombination processes of the charge carriers, hinders the diffusion process of the excitons, and quenches the excited states [165], [166], [167]. Thus, the charge carrier trapping process in the perovskite layers is one of the most important factors that affect the photon generation efficiency of PeLEDs.

**Figure 8:**
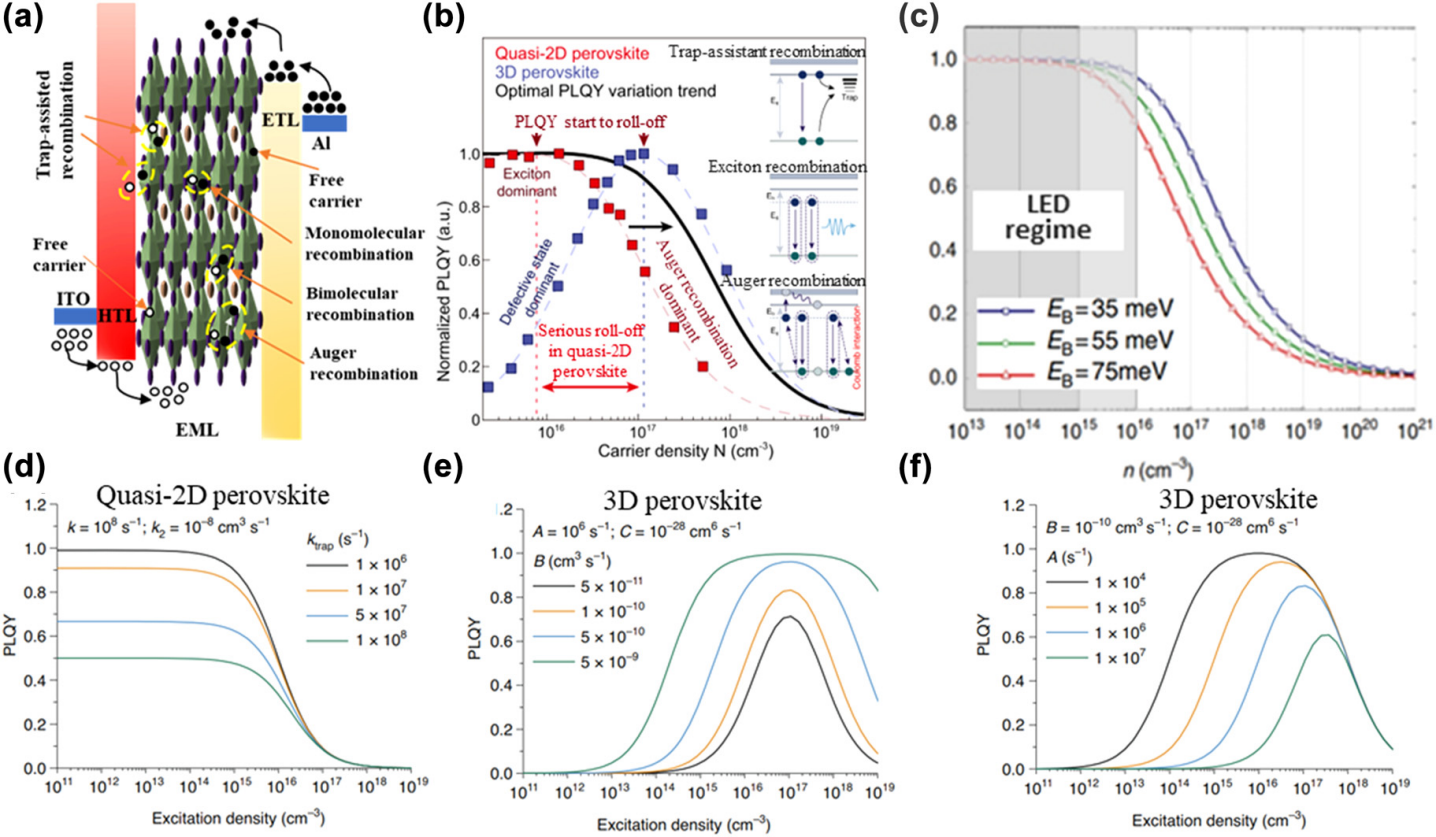
Charge carrier recombination, dynamics, and luminous efficiency. (a) Schematic diagram of charge carrier recombination in a PeLED device. (b) Effect of charge carrier recombination on PLQY in quasi-2D perovskite and 3D perovskite films. Reprinted with permission from Ref. [13]. Copyright 2021 Springer Nature. (c) Simulation of the free-charge fraction as a function of the total excitation density (*n*) at three different exciton binding energies at the thermal equilibrium according to Saha equation. Reprinted with permission from Ref. [172]. Copyright 2014 Springer Nature. PLQY-excitation density characteristics related to (d) varying trap-assisted nonradiative recombination rate, (e) varying nonradiative recombination coefficients, and (f) varying nonradiative monomolecular recombination coefficients. Panels d, e, and f reprinted with permission from Ref. [173]. Copyright 2021 Springer Nature.

Various traps in the perovskite layer hinder the charge carrier transport and also lead to a higher operation voltage. The injected carriers gradually fill the traps, and thus, one can observe a higher threshold voltage as well as a steeper increase in the current density and intensity of the emitted light [168, 169]. Trapping states have various origins and characters, and traps can be formed by the matrix materials as well as by the introduced emitter molecules. Charge-carrier recombination processes occur in the perovskite layer occurs as shown in Figure 8(a). At low excitation density, excitons directly dissociate into free carriers in the perovskite layer, thereby reducing the bimolecular radiative recombination. Due to the trap filling process, the injected/accumulated charge carrier is trapped to act as nonradiative recombination centers, leading to trap-assisted nonradiative recombination. An increasing excitation density suggests that the bimolecular radiative recombination rate is higher than the trap-assisted nonradiative recombination rate, indicating the dominance of the bimolecular radiative recombination. At a high excitation density, Auger recombination occurs, which acts as the dominant nonradiative recombination process as shown in Figure 8(b) [13].

Under an electrical bias, charge carriers (electrons and holes) are injected from the electrodes into the perovskite layer by drifting; these charge carriers are bound together by the coulombic attraction and form electron–hole pairs, i.e., excitons. EL occurs when the exciton undergoes a radiative, emitting photons and releasing energy. Thus, the balance of electrons and holes injected into the perovskite layer is crucial to maintain the luminance efficiency. In other words, when the optimal charge carrier balance is obtained, the electrons and holes generate the maximum possible number of photons in the recombination zone. Moreover, excess holes or electrons promote the migration of cations or anions that degrade the performance and stability of the PeLEDs [170].

At a low excitation density, trap filling processes occur, because of which the majority charge carriers can be trapped to attain the dynamic charge carrier balance. Figure 8(c) shows the simulation results of the free charge fraction as a function of a wide range of total excitation density based on the Saha–Langmuir equation, which indicates that efficient light emission in PeLEDs requires strongly bound electron–hole pairs [171, 172]. Moreover, the excessive charge carriers overflow from the perovskite layer to the transport layer by nonradiative processes without contributing to radiative recombination. Figure 8(d)–(f) illustrate the excitation-density-dependent the photoluminescence quantum yield (PLQY) with different recombination coefficients [173]. The imbalanced charge carriers also possibly enhance the Auger nonradiative recombination. Furthermore, because of the significantly different barriers for electron and hole injection, excessive charge carriers can accumulate at the HTL or ETL interface to form the extra built-in electrical field, which leads to the recombination zone shift. Local charge accumulation may degrade the interface between the perovskite layer and the transport layer, and defect states such as vacancies, interstitials, or antisites can also aggravate the degradation process [87]. The charge carrier trapping process at the interface generates a local electrical field, which deprotonates the organic cations and triggers an irreversible decomposition of the perovskite material [132, 140, 174].

### 5.4 Self-heating–induced degradation

The operation temperatures of the PeLED devices need to be carefully controlled. Even at a relatively low current density, the increase in the junction temperature caused by Joule heating substantially impacts the EQE and device stability. At a high current density, Joule heating is one of the crucial factors causing the EQE roll-off. The leakage current and nonradiative recombination (such as SRH recombination and Auger recombination) leads to energy loss in the PeLEDs, and this lost energy is converted into Joule heating, which increases the temperature and thus affects the performance and stability of the PeLEDs. The low thermal conductivity of the perovskites leads to the formation of a thermal dissipation barrier in the perovskite layer for heat accumulation. Joule heating is inevitably generated to increase the junction temperature during the device operation, and even a change of a few tens of degrees can also significantly affect the luminance efficiency and device stability. Accordingly to previous studies on the temperature dependence of EL in PeLEDs over a range of temperatures relevant to Joule heating, as the temperature increases, the EL gradually reduces until completely quenched [175, 176]. Ionic processes are extremely sensitive to temperature, and Joule heating significantly aggravates the ion-related effects, such as ion migration-induced generation/accumulation of defect states. In contrast, the trap-mediated nonradiative recombination enhances drastically with the temperature [89, 177]. The organic transport layer is thermodynamically unstable, and thus, the Joule heat generated during the device operation may irreversibly change the morphology of the transport layer. With the heat accumulation, the morphology of the TPBi film significantly changed, accompanied by the elements diffusing and the luminescence quenching at the perovskite layer [15]. Researchers have examined the effect of temperature on the device performance and stability through different experiments; for example, by tracking the EQE of a PeLED device at a constant current density at various temperatures [178]; by changing the excitation pulse width to maximize the EL intensity at a high current density [179]; and by altering the thickness of the perovskite layer to observe the effect of Joule heating dominance [178, 180].

**Figure 9:**
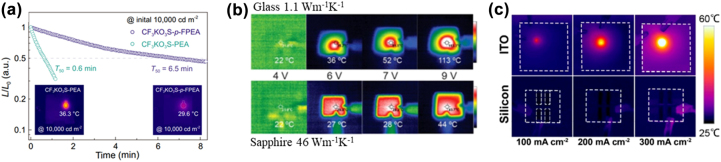
The effect of temperature evolution on performance and stability of PeLED devices. (a) Half-lifetime measurements and the corresponding spatial surface temperature of PeLEDs at an initial luminance. Reprinted with permission from Ref. [13]. Copyright 2021 Springer Nature. (b) Surface temperature distributions of PeLEDs grown on different substrates under different applied voltages. Reproduced with permission [50]. Copyright 2020, American Chemical Society. (c) Spatial heat distribution images of ITO-based and c–Si–based devices at different constant current densities. Reproduced with permission [176]. Copyright 2020, American Chemical Society.

Joule heating is considered as a major obstacle that impedes further improvements in the device performance and stability. When a PeLED device is driven at a high current density, the luminance increases, but it is typically accompanied by an increased heat generation. The increase in the temperature in turn causes a series of negative effects. The enhancement of the Joule heating effect decreases the luminance in the PeLEDs and results in the generation of more nonradiative recombination centers at the active layer. Consequently, the quantum efficiency decreases with the increasing temperature [181, 182]. The forward voltage drop is also related to the temperature increase due to a decrease series resistance and bandgap energy of the active region. The colors of PeLEDs also change with the increasing temperature. The shift toward longer wavelengths with lower energies, i.e., red shift, is due to the temperature dependence of the interparticle interactions induced by the energy gap shrinkage and electrical-field–induced Stark effect [183]. On the contrary, the shift toward shorter wavelengths with higher energies, i.e., blue shift, is due to the slow hot carrier relaxation and dynamic free charge carriers that fill the densities of states [184]. The degree of heat generation depends on the methods of heat dissipation. Previous reports have proposed several useful strategies to overcome the limitation of the thermal effect. For example, optimized the perovskite layer, doped the CTL, reduced the device area, used short-pulse electrical excitation, selected high-thermal conductivity substrates, and connected an external heat sink [183, 185]. The optimization of the perovskite layer can suppress Auger recombination, i.e., reducing the corresponding exciton binding energy; thus, the measured surface temperature decreases and improved the device stability as shown in Figure 9(a) [13]. The excellent heat sink was beneficial in reducing thermal accumulation within PeLED devices and enhanced luminance efficiency, as shown in Figure 9(b) and (c) [50, 176]. However, there is still debate as to which degradation mechanism (resistance or Auger recombination) induced Joule heating that eventually leads to device degradation at the high current region [89, 173, 186], and the uncertainty in the prediction of the junction temperature is because there are no direct ways to measure and evaluate the junction temperature. An in-depth understanding of the Joule heating effect on the performance and stability of PeLEDs is still lacking. The Joule heating effect of PeLEDs needs to demonstrate that there is no change during long-term stability analysis in order to enable correct correlation between luminance characteristics and temperature results. Unfortunately, the lack of accepted standards becomes a problem, and the experimental data of thermal tests are often of limited use in practice due to the accuracy questioned. Therefore, establishing standardization and improving experimental setups is an urgent issue.

### 5.5 Electrochemical reactions induced degradation

In addition to the above-mentioned physical mechanisms, various chemical reactions in PeLED devices initiated/caused by charges are also one of important reasons for device degradation. The anode material ITO is the most investigated electrode. The degradation of the ITO substrate not only changes the morphology and form voids but also leads to electrochemically induced reactions because of the diffusion of elements from ITO. The acidic nature of PEDOT:PSS causes corrosion of the ITO substrate and thus releases metallic elements that diffuse into the perovskite layer, resulting in defect formation [155]. These defects act as nonradiative recombination centers for the injected holes and electrons [187, 188]. In the cathode materials, the main electrochemical reaction of the electrodes is induced by water and oxygen [188, 189]. Reactions may occur even at the interface between the perovskite layer and the cathode. Electrochemical reactions alter the surface compositions and oxidation states at the perovskite layer/metal interfaces, lower the energy barrier at the exposed contacts, and provide a source for the diffusion of the defect states [190, 191]. Electrochemical reactions within the PeLEDs are also possible. There was ample evidence that the ion migration-related electrochemical instability induces PeLEDs degradation under an electrical bias, and the corresponding reaction products, such as exciton quenchers or charge carrier traps, act as nonradiative recombination centers [21, 192], [193], [194]. The increased iodide ratio in TFB hole injection layer of 3D-PeLEDs causes performance degradation, and decrease in EQE can be largely derived from the destruction of TFB hole injection layer via electrochemical reaction [192]. The excess KI additive in PeLEDs leads to the significant reduction of EQE roll-off because iodide and iodine produced by electrochemical reactions serve as an additional EL quenching pathway [133, 195, 196]. Electrochemical reactions lead to a decomposition of perovskite into PbI_2_ at the anode interface and Pb^2+^ is reduced to Pb^0^ at the cathode interface, due to ion migration and compensation of electrons [77, 142, 197]. Iodine anions interact with the Ag electrode to create an insulating compound AgI at an electrical bias condition [153]. Electrochemical reactions between bromide anions and Ag electrode produce an insulating compound AgBr in MAPbBr_3_ [163].

## 6 Conclusions and perspectives

In this paper, we reviewed and categorized the known degradation processes and associated mechanisms that affect the performance of PeLEDs. Development of PeLEDs has made significant progress in the past few years, leading to low-cost fabricating methods and high luminance efficiencies. However, the stability problem caused by the device degradation still hinders the widespread application of PeLEDs.

The knowledge and understanding of degradation mechanisms due to physical changes and chemical reactions during device operation in a relatively short time have improved significantly, based on the extensive experience and knowledge accumulated through inorganic and organic LED technology research conducted in various fields from material chemistry to device engineering. Although many degradation mechanisms have similar characterization results, they can still be classified according to various factors that cause device degradation. We systematically elucidated the degradation mechanisms of PeLEDs under an electrical bias in terms of external and internal factors, such as process parameters, environmental factors, charge carrier kinetics, recombination quenching mechanisms, thermodynamics, and chemical reactions.

We also reviewed the most common analytical techniques suitable for investigating the degradation phenomena in PeLEDs. L–I–V curve analysis and impedance spectroscopy are the most commonly used analytical techniques to analyze the physical changes and determine the degradation processes and associated mechanisms. X-ray techniques are most commonly employed for analyzing the chemical reactions and identifying the degradation products. However, due to the influence of perovskite material properties and device structure, many measurement results cannot accurately explain the degradation mechanisms of PeLEDs and provide only qualitative conclusions for reference. Moreover, the physical quantities measured in optoelectronic characterizations are rarely directly related to the desired quantities. For example, in the case of L–I–V characteristic measurements, the analysis is based on injection current, bias voltage, and luminance. In many instances, the desired parameter can be derived via the physical model and mathematical relationship between the measured and desired quantities. The nonradiative current as the desired quantity, which can be separated from the injection current as the measured quantity, is used to analyze the carrier transport and device degradation in detail. Separating the injection current components is a considerable challenge, indicating that a more in-depth understanding of the device properties, such as carrier transport processes, luminous efficiency, and device degradation, is required. In addition, the role of Joule heating played in the PeLED device is also an important point of contention. Various degradation processes under a bias voltage generally coexist, and thus, the identification of the ultimate degradation mechanism is difficult. As a result, the contribution of Joule heating cannot be properly disentangled and explained. Further, the insufficient knowledge of the temperature change inside the device disputes the thermal-induced device degradation mechanism. It is generally well known that the most intuitive investigation of the Joule heating effect is the temperature change. However, thus far, known measurement methods have not been widely recognized because of their own limitations. Hence, researchers should be encouraged to study the temperature measurement methods of inorganic and organic LEDs and develop an analytical technique that can be employed to investigate the Joule heating effect of PeLEDs.

Some fabrication strategies that significantly improve the performance and stability of PeLEDs are well known. The use of highly purified materials and substrates, optimized deposition processes, and appropriate packaging technology generally result in a better device performance and stability. Although the effects of extrinsic factors, such as moisture and oxygen, on the perovskite remain controversial, more evidences indicate that moisture and oxygen can destabilize the PeLED devices. Most internal degradation processes of the PeLEDs can be summarized as correlated defect behavior, although the perovskite materials have shown remarkable tolerance to defects, and high-density defects mediated carrier transport processes are the dominant causes of the inefficiency and instability of PeLEDs. Some chemical design guidelines for PeLED materials are based on certain features of the chemical structure of the associated defect behaviors. For example, doping and constructing a core–shell structure to inhibit ion migration and improve charge carrier injection and transport, and utilizing passivation strategies to enhance defect management for improving the performance and stability of PeLEDs. While the strategies of defect management suppress the defects to a certain extent, various effective strategies of defect suppression are still needed to further improve the device performance and stability. In PeLEDs, the effect of defects is less severe because most of the intrinsic defect states lie within the bands or are close to the bandgap; however, it does not indicate that defects are unimportant. Defects play a key role in limiting the efficiency of electrical to photon energy conversion by nonradiative recombination. Defect-induced nonradiative recombination depends on the density, energetic position, and location of the defects. To deduce the accurate defect characteristics of PeLEDs, both theoretical and experimental analyses are essential. However, the study on the conditions and mechanisms of defect formation is far behind that on strategies of defect control. The effect of defects on PeLEDs has rarely been reported, and there is a lack of understanding of the defect state under the device operation state. Therefore, an in-depth investigation of defect-induced degradation mechanisms through advanced characterization experiments is imperative to provide guidelines for effective defect suppression strategies.

Undoubtedly, the investigation of degradation mechanisms is crucial for the development of PeLEDs. We reviewed the various reasons that may cause the degradation of PeLEDs and attempted to correlate the degradation mechanisms to the changes in the physical structure or chemical composition. Separation of the various changes into external and internal factors is necessary to better elucidate the origin of device inefficiency and instability. For example, the correlation of process parameters, device performance, and stability; the influence of passivation and post-treatment techniques; the erosion of device structure by moisture and oxygen; the intermediates and final products formed during the degradation processes; the generation mechanism of nonradiative recombination centers; the effect of ion migration on each functional layer; and the Joule heating effect inside the PeLED structure.

We anticipate that this review will encourage more researchers to study the issues related to PeLED degradation, which will be instrumental for the development of PeLEDs with high efficiencies and long lifetimes.
